# Using Remote Technology to Engage Patients with Interstitial Lung Diseases in a Home Exercise Program: A Pilot Study

**DOI:** 10.3390/life14020265

**Published:** 2024-02-17

**Authors:** Antonio Sarmento, Kaitlin King, Diana C. Sanchez-Ramirez

**Affiliations:** Department of Respiratory Therapy, University of Manitoba, Winnipeg, MB R3E 0T6, Canada; antonio.sarmentodanobrega@umanitoba.ca (A.S.); kaitlin.king@umanitoba.ca (K.K.)

**Keywords:** interstitial lung diseases, feasibility, compliance, virtual exercises

## Abstract

Introduction: The access and compliance of patients with interstitial lung diseases (ILDs) to exercise programs (EPs) remain challenges. Objectives: We assessed the dropout rate, intervention completion, compliance with data acquisition and submission, safety, and satisfaction of a home EP delivered via video conference (EP_VC_ group) or self-directed (EP_SD_ group) to patients with ILD. Pre- and post-intervention changes in patient outcomes (dyspnea, fatigue, exercise capacity, lung function, and quality of life) were secondarily explored. Material and Methods: Groups performed an eight-week virtual EP three times/week. Video conferences were led by a registered respiratory therapist, whereas self-directed exercises were completed following a pre-recorded video. Participants submitted spirometry, heart rate, and SpO_2_ results weekly to the research team. Results: Fourteen patients with ILD were equally assigned to the EP_VC_ and EP_SD_ groups, but three from the EP_SD_ group dropped out after the initial assessment (dropout rate of 42.8% in the EP_SD_ group). Eleven patients (mean age of 67 ± 12 years) completed 96.5% of sessions. Compliance with data acquisition and submission was optimal (≥97.6% in both groups), and no adverse events were reported. Changes in overall fatigue severity were significantly different between groups (*p* = 0.014, Cohen’s r = 0.64). Conclusions: The results suggest that a structured virtual EP delivered via video conference or pre-recorded video can be feasible, safe, and acceptable for patients with ILD.

## 1. Introduction

Interstitial lung diseases (ILDs) comprise a group of disorders characterized by impaired lung function, high levels of dyspnea and fatigue, reduced functional capacity, and low quality of life [1,2]. Clinically important improvements in health outcomes and survival have been found in patients with ILD who participated in pulmonary rehabilitation programs with exercise training [3,4,5]. Despite the multiple benefits, patients with chronic lung diseases frequently face difficulties in accessing and completing exercise programs (EPs) [6,7,8]. In this context, home-based exercises remotely delivered using technology have emerged as a promising alternative to facilitate the participation and compliance of patients with ILD in rehabilitation programs. Individualized training programs tailored to specific individual needs can also be provided to groups of patients to enhance compliance [9]. Therefore, we conducted a pilot study to assess the dropout rate, intervention completion, compliance with data acquisition and submission, safety, and satisfaction of patients with ILD who performed a home EP delivered via video conference or self-directed (with a pre-recorded video). Secondarily, changes in patient outcomes (dyspnea, fatigue, exercise capacity, lung function, and health-related quality of life [HRQoL]) were explored.

## 2. Material and Methods

### 2.1. Design and Participants

A two-group randomized pre- and post-test study (ClinicalTrials.gov ID NCT04946708 and research ethics committee of the University of Manitoba HS24936 B2021:051) was performed with a convenience sample of adults with ILD (diagnosed by a respirologist) who had access to a smartphone or tablet and home internet; individuals should not have been involved in any structured physical activity program. Those with acute exacerbation of the condition requiring care at an emergency department or hospitalization, pre-existing medical limitations to engaging in light-intensity physical activity (e.g., neurological conditions, severe orthopedic conditions, pending hip/knee replacement, dementia, or chronic vertigo), history of falls over the past year, inability to ambulate three blocks independently without supervision, severe hearing or visual impairments that would inhibit remote communication with the research team (e.g., video conference), or inability to complete basic tasks on a smartphone or tablet were excluded. All participants signed the informed consent form.

Inclusion criteria were assessed during a screening call, then participants were randomly assigned (www.randomlists.com, accessed on 1 September 2021) to the video conference (EP_VC_) or self-directed (EP_SD_) exercise group. The informed consent form and details about the EP were sent via e-mail according to the assigned group, whereas the following documents and equipment were delivered to the home of each participant: one digital finger oximeter (LOOKEE^®^, New York, NY, USA), one portable spirometer (SpiroBank Smart spirometer, MIR, Rome, Italy), one nose clip, three disposable mouthpieces, one activity diary, printed versions of questionnaires and the exercise program, and one prepaid envelope for returning the equipment and diary after completing the home EP. Subsequently, a registered respiratory therapist (RT) scheduled an individual virtual appointment via video conference (Zoom) to clarify and sign the informed consent form; provide education and personalized recommendations regarding maximum heart rate (HR) and minimum oxygen saturation (SpO_2_) during exercise [10,11]; explain study procedures, instruments, and equipment; collect demographic information; and complete the initial assessment.

### 2.2. Exercise Program

An eight-week EP comprising warm-up, resistance and aerobic exercise, and cool-down phases (Table 1) was designed by an RT and a physical therapist (PT) based on ATS/ERS recommendations [12] and recent reviews [9,13]; resistance exercises were performed incorporating household items such as canned food. Participants assigned to the EP_VC_ group engaged in a 30 min group EP conducted via video conference (Zoom) three times a week. In each session, they could also exchange information or engage in informal interactions with other group members for 15 min (5 min pre- and 10 min post-exercises). The RT led all sessions, addressed general questions from participants, and discussed patient-specific exercise modifications with the PT if necessary. In the EP_SD_ group, participants were instructed to independently perform the same exercise program three times a week using a pre-recorded video created by the research team (PT and RT) and uploaded on YouTube. Participants of this group were also advised to pause the video while (i) doing additional repetitions of the exercise based on their previous performance (increasing demand) or (ii) resting (decreasing demand) and to continue with the next exercise when they were ready.

Following the recommendations provided during the initial assessment, participants of both groups were required to submit acceptable spirometry results to the team weekly, and HR and SpO_2_ values were to be recorded in the diary before and after each exercise session. The RT called participants once a week to address inquiries and monitor symptoms and could be contacted via e-mail or phone any time during the study for questions or concerns. Adjustments to training intensity (e.g., number of repetitions) were completed during the video conference sessions (EP_VC_) or through the weekly phone calls (EP_SD_) according to symptoms and exercise tolerance of the patients.

### 2.3. Feasibility

The feasibility components studied included dropout rate, intervention completion, compliance with data submission, safety, and satisfaction. The dropout rate represented the proportion of individuals who discontinued the rehabilitation after randomization and before completing 80% of the sessions due to adverse events or personal preferences [14,15], whereas intervention completion was calculated as the proportion of sessions attended by patients [16,17]. The number of sessions completed by participants was recorded by the RT who led the group sessions (EP_VC_) or by the patient in a diary (EP_SD_). Compliance with data submission was determined as the proportion of data sent by patients to the research team. Safety was assessed by the occurrence of adverse events associated with participation in the EP, while satisfaction with the program was collected after the EP using a scale ranging from 0 (low) to 10 (high).

### 2.4. Assessments

The initial and final assessments were conducted virtually. Sex (female, male, or other), age, height, weight, smoking history (yes or no), self-reported physical activity level (low, moderate, or active), and use of prescribed supplementary oxygen were collected only in the initial assessment. Secondary outcomes (dyspnea, fatigue, exercise capacity, lung function, and generic and ILD-specific health-related quality of life) were collected in the initial and final assessments.

Dyspnea was assessed with the modified Borg scale (0 to 10) [18]. The severity and frequency of fatigue in everyday life were evaluated with the Fatigue Severity Scale (FSS) using a 7-point Likert scale ranging from 1 (strongly disagree) to 7 (strongly agree). Higher scores represent more severe fatigue and a higher impact on the activities of the patient [19,20]. This questionnaire also had a visual analog scale that assessed overall fatigue severity; scores ranged from 0 (worst) to 10 (normal).

Exercise capacity was evaluated with the one-minute sit-to-stand test. Participants were asked to sit and stand on a stable armless chair as many times as they could at their own pace for one minute; the maximum number of repetitions was recorded [21,22]. HR and SPO_2_ were measured before and after the test using a digital pulse oximeter.

Spirometries were conducted to assess lung forced vital capacity (FVC), forced expiratory volume in the first second (FEV_1_), FVC/FEV_1_, and peak expiratory flow. Participants completed three valid trials using the portable spirometer and associated MIR SpiroBank app during initial and final assessments, as well as once a week during the study, and sent the results via e-mail or text messages to the research team. Tests followed ATS/ERS recommendations [23], and predicted values are reported according to the Canadian population [24].

Generic and ILD-specific HRQoLs were assessed using the EuroQol-5 Dimensions-5 Levels (EQ-5D-5L) [25] and King’s Brief Interstitial Lung Disease (KBILD) [26], respectively. The former addresses five domains (mobility, self-care, usual activities, pain/discomfort, and anxiety) using a 5-point scale, and total scores (EQ-5D-5L index) were calculated following the value set for the Canadian population (the higher the score, the worse the HRQoL). The visual analog scale (0 to 100) of the EQ-5D-5L was also used to assess the current health state of participants (higher values represent better health). The KBILD is a self-completed health status questionnaire specific to ILD that comprises 15 items divided into 3 domains (breathlessness and activity, chest symptoms, and psychological impact); responses are provided on a 7-point Likert scale. Total and domain scores range from 0 to 100, and higher values indicate better health [27].

## 3. Statistical Analysis

Data are presented as absolute and relative frequencies or median and 25–75% interquartile range (IQR_25–75%_). Median changes in dyspnea, fatigue, exercise capacity, lung function, and HRQoL were compared between the EP_VC_ and EP_SD_ groups using the Mann–Whitney test to explore potential improvements in outcomes. Cohen’s r effect size was also calculated for variables that were significantly different [28]. Statistical analyses were performed using the Statistical Package for Social Sciences, version 28 (IBM Corp., San Francisco, CA, USA). Significance was set at *p* < 0.05.

## 4. Results

**Figure 1:**
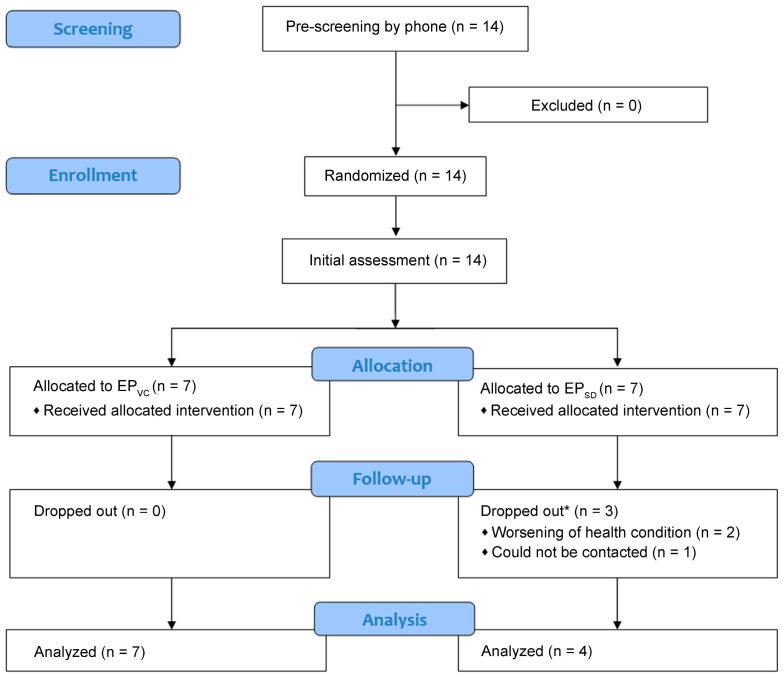
Flowchart of participant recruitment and intervention. EP_VC_: exercise program via video conference; EP_SD_: self-directed exercises. * Dropped out after the first week of intervention.

Fourteen patients were recruited, consented to participate in the study, and were randomly assigned to the EP_VC_ group (seven patients) or the EP_SD_ group (seven patients). All participants performed the initial assessment, but three from the EP_SD_ group dropped out after the first week of the program: two due to worsening of their health condition that required additional medical treatment (not related to the exercise program), and one could not be contacted (dropout rate of 0% in the EP_VC_ group, 42.8% in the EP_SD_ group, and 21.5% in the total sample) (Figure 1). Therefore, the final sample comprised 11 patients (mean age of 67 ± 12 years, mean height of 173.3 ± 12 cm, and mean weight of 83.3 ± 14 kg; 57% males): 7 in the EP_VC_ group and 4 in the EP_SD_ group. Five patients (four in the EP_VC_ group, one in the EP_SD_ group) used prescribed supplemental oxygen therapy. Four participants in the EP_VC_ group and two in the EP_SD_ group self-reported an active level of physical activity (Table 2). Participants completed 96.5% of the exercise sessions (intervention completion rate was 98.2% in the EP_VC_ group and 94.8% in the EP_SD_ group).

Compliance with submission of spirometry results was 98.2% and 100% in the EP_VC_ and EP_SD_ groups, respectively, whereas compliance with HR and SpO_2_ data submission was 97.6% in the EP_VC_ group and 98.2% in the EP_SD_ group. No adverse events were observed, and participants in both groups were very satisfied with the program (9, IQR_25–75%_: 9; 10).

There were no significant pre–post improvements in dyspnea, fatigue, exercise capacity, lung function, and HRQoL in either group. FSS_VAS_ changes were significantly different between the EP_VC_ and EP_SD_ groups (*p* = 0.014, Cohen’s r = 0.64) (Table 3).

## 5. Discussion

The results of this pilot study suggested that an eight-week EP delivered via video conference or self-directed was feasible and safe for patients with ILD. Intervention completion rate, compliance with data submission, and satisfaction with the program among participants were high in both groups. Although patient outcomes did not improve significantly, likely explained by the small sample size, the results indicated that these technology-aided approaches could facilitate the delivery of home EPs for patients with ILD.

The increasing development of technology presents a promising resource to facilitate the remote delivery of EPs, addressing several challenges of in-person programs, including mobility restrictions, distance, access, and participation [29,30]. This is important because in-person pulmonary rehabilitation programs are estimated to be underused in many countries [8,31,32,33,34], which may affect the exercise capacity and influence the survival of patients with chronic lung diseases [35,36,37]. Most studies with a larger number of patients with ILDs have also reported intervention completion rates lower than 80% [38,39,40]. Therefore, there is an urgent need for feasible approaches that ensure the proper delivery of exercises with high patient participation [41]. In this pilot study, participants in the EP_VC_ group mentioned the social interaction with peers during the intervention as a positive aspect of the program. Although the dropouts observed in the virtual EP_SD_ group were apparently unrelated to the program, they could have been influenced by a lack of motivation as no peer support or direct supervision was provided during the EP_SD_ sessions. Nevertheless, one participant in the EP_SD_ group valued the flexibility of the program (patients could perform exercises in the preferred environment at any time). As patient engagement and acceptance are crucial for the success of virtual rehabilitation [42], incorporating support, motivational strategies, and exercise variations in self-directed virtual programs might help improve and maintain patient motivation [43,44,45]. In general, if possible, it would be valuable to consider personal preferences and needs when designing and choosing the right virtual program for each participant [46].

The COVID-19 pandemic accelerated the adoption of remote rehabilitation approaches for patients with ILDs [45,47,48], but virtual assessments and interventions are still challenging. Variability in technology literacy is common [45], especially among older adults who may be hesitant to use online applications or platforms to exercise on their own [49]. Participants in our study were mostly older adults; some needed step-by-step guidance by phone to access the Zoom platform, while others took longer than expected to understand and set up the apps during initial assessment. Nevertheless, there were no major technical difficulties or complaints during the development of the program or the final assessment. In general, the components of our virtual EP approaches were well received by participants. Moreover, participation (i.e., mean of 23 out of 24 sessions) and satisfaction with the program were optimal, and no safety issues were reported. Despite the small sample size, satisfaction levels corroborated those found in other studies [50,51]. Participation was higher than observed in in-person [52,53] and virtual pulmonary rehabilitation programs [54,55,56,57] with exercise components for patients with chronic lung diseases, possibly because our virtual EP was simple to understand and perform (i.e., no technological issues), individually tailored, and accessible [44]. We also observed that the group sessions via video conference may have had a better effect on perceived fatigue compared with the self-directed group. Although similar results related to changes in fatigue scores after a remote home-based EP were observed in a recent cohort of patients with ILD [58], more research is required to confirm this finding and its possible causes.

Regular assessment of lung function and pulse oximetry is valuable for the overall care of patients with ILD, including the prediction of exacerbation signs and self-pacing during exercises, and it improves the communication between patients and care providers [59,60,61]. In our study, the portable devices provided to participants were easy to use, which may have influenced the high compliance with data gathering and submission observed during the program. In addition, evidence indicates that home spirometry is a valid and relatively new monitoring tool in the management of ILD patients [62,63,64] that, together with the recent integration of mobile and online health applications into chronic lung disease assessment and management, has the potential to reduce the length of waiting lists and costs and improve patient self-monitoring and follow-up. At the same time, WiFi use enables data collection and sharing [50,59,65,66,67]. From the health professional perspective, the high adherence of patients with remote data sharing during home EPs may ensure safer exercises at appropriate intensities, provide direct readings without relying on participant interpretation, reduce costs, and help monitor exercise progression, symptoms, and signs of exacerbation [59,68,69].

Although the effects of the intervention on patient outcomes were secondarily explored, we were aware that the sample size was too small to reach statistical significance. However, this pilot study was designed to explore the feasibility, safety, and satisfaction of patients with ILD, and it was beyond our scope to recruit a larger sample to assess the efficacy of the program. A control group was not included since evidence has already established that the effects of remote programs are superior to no rehabilitation and similar to in-person rehabilitation [70]. Only one video was provided to patients assigned to the EP_SD_ group; therefore, further studies should develop and evaluate the effect of multiple exercise videos with various intensity levels to avoid monotony and facilitate exercise progression. Some alternatives to peer support and direct supervision should also be explored in this group. Lastly, most participants from the EP_VC_ group reported being physically active, and physical activity habits were not controlled during the study, which may have influenced the effects of the EP. Despite these limitations, and different from other remote or home-based programs that usually completed the assessments in person, we conducted a completely remote home EP, including initial and final assessments, that was safe and well received by participants [71,72]. This is relevant in light of the pandemic-acquired use of technology by patients with ILD, which may have expanded their acceptability, skills, and attitudes toward telehealth, thus potentially facilitating their access to virtual exercise and pulmonary rehabilitation programs [47]. In addition, this study strengthens the emerging evidence supporting the feasibility of virtually delivered exercise programs for patients with ILD [15,71,73]. In this context, future studies with a larger number of patients with similar physical activity levels are needed to (i) explore the impacts and long-term effects of EPs and (ii) determine whether virtual assessments and delivery of virtual home-based EPs could work as an add-on or partially substitute the components of in-person PR for patients with ILD.

## 6. Conclusions

The results of this study suggested that a structured virtual EP delivered via video conference or pre-recorded videos can be feasible, safe, and acceptable for patients with ILD in a real-world setting. These approaches may facilitate the delivery of home-based exercises and increase patient access, participation, and compliance. Larger studies are needed to explore their clinical effectiveness and long-term effects in patients with ILD.

## Figures and Tables

**Table 1:** Pulmonary rehabilitation exercise program. Exercises were performed with participants from the EP_VC_ and EP_SD_ groups seated on a chair initially. They were encouraged to use household items for upper limb exercises, and progressed from sitting to standing position when the participant and the therapist deemed it appropriate.

Exercises	Repetitions	Sets	Hold Time
Warm-up			
Head turns	10	1 each side	2 s
Chin-to-chest	10	1	2 s
Dynamic arm stretches			
Seated quadratus lumborum	2	1 each side	5 s
Posterior shoulder capsular	1	1 each side	5 s
Triceps	1	1 each side	5 s
Shoulder rolls	10	2 (forward and backward)	2 s
Trunk rotation	10	1 each side	2 s
March	10	2	2 s
Leg stretches	2	1 each side	5 s
Resistance exercises			
Biceps curls	10	2	2 s
Side arm raises	5	2	2 s
Heel raises	10	1	2 s
Knee extension	5	1 each side	5 s
Bicycle crunch	10	1	2 s
Aerobic exercises			
Forward arm punches	10	1	2 s
Jumping jacks	5	2	2 s
Sit-to-stand	10	1	2 s
Cool down (stretches)			
Neck stretch	5	1 each side	5 s
Posterior shoulder capsular	2	1 each side	5 s
Wrist rotation	2	1 each side	5 s
Wrist flexor extension	2	1 each side	5 s
Chest	2	1 each side	5 s
Seated quadratus lumborum	2	1 each side	5 s
Calves	2	1 each side	5 s
Hamstrings	2	1 each side	5 s

**Table 2:** Characteristics of participants.

	EP_VC_ (*n* = 7)	EP_SD_ (*n* = 4)
Sex		
Male	5 (71.4)	2 (50)
Female	2 (28.6)	2 (50)
Age, years	75 (69–77)	63 (42–69)
Height, centimeters	180 (168–185)	164 (156–178)
Weight, kilograms	86 (82–100)	74 (60–93)
Smoking history		
Yes	5 (71.4)	4 (100)
No	2 (28.6)	0 (0)
Physical activity level		
Low	2 (28.6)	1 (25)
Moderate	1 (14.3)	1 (25)
Active	4 (57.1)	2 (50)
Oxygen		
Yes	4 (57.1)	1 (25)
No	3 (42.9)	3 (75)

Data shown as absolute (*n*) and relative frequency (%) or median and 25–75% interquartile range. EP_VC_: exercise program via video conference; EP_SD_: self-directed exercises.

**Table 3:** Secondary outcomes pre- and post-exercise program.

	EP_VC_ (*n* = 7)			EP_SD_ (*n* = 4)		
	Pre	Post	Δ_post-pre_	Pre	Post	Δ_post-pre_
Dyspnea	2 [0.5; 2.5]	1 [0; 2]	0 [−1.5; 0.5]	2.5 [0.5; 3]	1.2 [0.1; 2.7]	0 [−1.8; 0]
FSS_total score_	34 [26; 32]	41 [21; 48]	0 [−4; 7]	34.5 [21.7; 45]	25.5 [15.7; 2.2]	−4.5 [−12.7; −1.5]
FSS_VAS_	4 [2; 7]	7 [6; 8]	2 [0; 4]	6 [4.25; 8.5]	3.5 [41.2; 7.7]	−2 [−3; −0.2] *
Exercise capacity	22 [17; 29]	23 [20; 27]	1 [−3; 2]	29 [17; 32]	27 [18.5; 30.2]	0.5 [−5.5; 2.7]
Lung function, %pred						
FVC	67 [47.2; 87.4]	76.1 [66.9; 91.1]	2.4 [−0.9; 29]	85.3 [55.7; 96.6]	87.3 [71.2; 101]	6.7 [−5.3; 26]
FEV_1_	80.6 [43.2; 91.6]	81.9 [74.8; 95.6]	4 [−5.8; 38]	89.8 [52.8; 99.5]	86.3 [73.4; 108]	8.7 [−9; 21]
FEV_1_/FVC	101.9 [92; 106.9]	105.8 [101; 109.9]	3 [0; 5.9]	100 [93.3; 107]	102.9 [96.5; 107]	1.4 [−4.8; 9.3]
PEF	88.2 [77.7; 123]	106 [95; 126]	17 [−6.7; 18.2]	108 [70.7; 238]	101 [82; 141.9]	−6.6 [−99; 14.4]
EQ-5D-5L						
Mobility	1 [1; 2]	1 [1; 1]	0 [0; 0]	1 [1; 1.7]	1 [1; 2.5]	0 [0; 0.7]
Self-care	1 [1; 1]	1 [1; 1]	0 [0; 0]	1 [1; 1]	1 [1; 1]	0 [0; 0]
Usual activities	2 [1; 3]	1 [1; 2]	0 [−1; 0]	1.5 [1; 2]	1 [1; 1.7]	0 [−0.7; 0]
Pain/discomfort	2 [1; 2]	2 [1; 2]	0 [−1; 0]	2.5 [2; 3.7]	1.5 [1; 2.7]	−1 [−1.7; −0.2]
Anxiety	1 [1; 2]	1 [1; 2]	0 [0; 0]	2 [1.2; 2.7]	1 [1; 1.7]	−0.5 [−1.7; 0]
EQ-5D-5L index	0.87 [0.79; 0.93]	0.91 [0.87; 0.93]	0 [−0.03; 0.44]	0.78 [0.65; 0.89]	0.91 [0.80; 0.95]	0.06 [0.04; 0.23]
EQ_VAS_	80 [50; 90]	85 [70; 85]	5 [−5; 10]	72.5 [55; 82.5]	75 [51.2; 87.5]	−5 [−5; 13.7]
KBILD						
Psychological	78.6 [64.3; 92.9]	83.3 [59.5; 92.9]	−2.3 [−4.8; 17]	85.7 [53; 95.2]	79.7 [54.7; 92.3]	5.9 [−20; 7]
BA	70.8 [58.3; 75]	70.8 [66.7; 83.3]	4.2 [−4.2; 12]	66.6 [43.7; 89.5]	77.1 [50; 88.5]	6.3 [−14; 24]
Chest symptoms	77.8 [61.1; 88.9]	77.8 [77.8; 88.9]	5.6 [0; 11.1]	86.1 [58.3; 93]	75 [41.6; 91.6]	−8.3 [−25; 4]
Total score	70.4 [66.8; 87.9]	78.4 [69.4; 86.8]	1.4 [−3; 11.6]	79.5 [52.1; 92.2]	77.6 [48.8; 90.4]	1.2 [−20; 11]

Data shown as 25–75% interquartile range. EP_VC_: exercise program via video conference; EP_SD_: self-directed exercises. Δ_post-pre_ represents median changes between post- and pre-EP values. FSS: fatigue severity score; FSS_VAS_: FSS visual analog scale; %pred: percentage of predicted values; FVC: forced vital capacity; FEV_1_: forced expiratory volume in the first second; PEF: peak expiratory flow. EQ-5D-5L: EuroQol-5 Dimensions-5 Levels; EQ_VAS_: EuroQol visual analog scale; KBILD: King’s Brief Interstitial Lung Disease; BA: breathlessness and activities domain. * *p* < 0.05 in Δ_post-pre_ between EP_VC_ and EP_SD_.

## Data Availability

Data presented in this study are available on request from the corresponding author.
